# Person, Affective, Cognitive, and Executive Dimensions Associated with Problematic Social Media Use, Internet Gaming Disorder, and Their Co-Occurrence in Young Adults

**DOI:** 10.3390/ejihpe16090141

**Published:** 2026-09-15

**Authors:** Melany León-Méndez, Iván Padrón, Ascensión Fumero, Lilisbeth Perestelo-Pérez, Rosario J. Marrero

**Affiliations:** 1 Instituto Universitario de Neurociencia, Universidad de La Laguna, 38200 Tenerife, Spain; 2 Department of Clinical Psychology, Psychobiology, and Methodology, Universidad de La Laguna, 38200 Tenerife, Spain; 3 Department of Developmental Psychology and Education, Universidad de La Laguna, 38200 Tenerife, Spain; 4 Evaluation Unit (SESCS), Canary Islands Health Service (SCS), Canary Islands Health Research Institute (IISC), Network for Research on Chronicity, Primary Care, and Health Promotion (RICAPPS), The Spanish Network of Agencies for Health Technology Assessment and Services of the National Health System (RedETS), 38109 Tenerife, Spain

**Keywords:** problematic social media use, internet gaming disorder, I-PACE model, young adults, personality traits, emotion regulation, executive functioning

## Abstract

**Background**: Problematic Social Media Use (PSMU) and Internet Gaming Disorder (IGD) are online behaviors associated with adverse psychosocial outcomes in young adults. However, person, affective, cognitive, and executive correlates of these behaviors remain insufficiently examined. This study examined I-PACE (Interaction of Person–Affect–Cognition–Execution) model dimensions associated with PSMU and IGD, and their co-occurrence in young adults from dimensional and categorical perspectives. **Methods**: A total of 1586 students (68.9% female; M = 20.42 years, SD = 2.41) completed self-report measures assessing PSMU, IGD, personality traits, emotion regulation difficulties, decision-making styles, and executive functioning. **Results**: Overall, 17.97% screened positive for PSMU, 16.02% for IGD, and 10.65% for both behaviors. In the entire sample, PSMU and IGD were both related to lower conscientiousness, difficulties in goal-directed behavior, non-acceptance of negative emotions, and emotional and executive control deficits. PSMU was additionally related to extraversion, limited access to effective emotion regulation strategies, and dependent decision-making style, whereas agreeableness, impulse control difficulties, and social behavior control deficits were related to IGD. From a categorical perspective, executive deficits were associated with higher PSMU symptomatology, whereas lower agreeableness and conscientiousness, greater difficulties in goal-directed behavior, and a lower tendency to use an intuitive decision-making style were related to higher IGD symptomatology. Among participants with co-occurring PSMU and IGD, lower agreeableness, lower openness to experience, and greater difficulties in goal-directed behavior were associated with higher PSMU and IGD symptoms. Dependent decision-making was additionally associated with higher PSMU symptoms. Furthermore, mean comparisons revealed that the PSMU and IGD co-occurring group showed higher levels of neuroticism, emotion dysregulation, spontaneous/avoidant decision-making styles, and executive, emotional, and social behavior control deficits than single-behavior groups. **Conclusions**: The findings suggest partially distinct patterns of association between person, affective, cognitive, and executive dimensions, depending on whether problematic online behaviors were considered from a dimensional or categorical perspective. These exploratory results require longitudinal replication and validation in clinical samples and in samples from other cultures, or from different developmental stages.

## 1. Introduction

Internet use has increased substantially worldwide, particularly among adolescents and young adults (Meng et al., 2022). Among the most prevalent forms of behavioral addictions are Internet Gaming Disorder (IGD) and Problematic Social Media Use (PSMU) (Geisel et al., 2021). According to the Spanish Observatory on Drugs and Addictions (OEDA, 2025), 15.3% of adolescents show signs of possible PSMU, and 3.5% meet the criteria for IGD. Although these behaviors share several characteristics, including psychopathological symptoms, cognitive correlates, and sociodemographic factors (Philippi et al., 2024), conceptualizing behavioral addictions as a homogeneous category may obscure important differences between specific problematic online behaviors (Baggio et al., 2022).

Gaming disorder is recognized in the ICD-11 as a persistent gaming behavior associated with clinically significant impairment (WHO, 2022), whereas Internet Gaming Disorder (IGD) is included in the DSM-5 as a condition that requires further study before being considered a disorder (American Psychiatric Association, 2022). On the other hand, PSMU has been consistently associated with psychosocial difficulties despite not yet being included in major diagnostic manuals. Both behaviors have been linked to poor academic performance, anxiety, depression, impaired self-control, and alterations in brain functioning (Berloffa et al., 2022; León-Méndez et al., 2024; Tian et al., 2023). Moreover, the co-occurrence of PSMU and IGD appears to be associated with greater psychological distress than either behavior alone (M. Müller & Montag, 2017; Pontes, 2017; Strizek, 2022).

The Interaction of Person–Affect–Cognition–Execution (I-PACE) model (Brand et al., 2019, 2025), which was developed from previous explanatory models, proposes that behavioral addictions develop through interactions among predisposing person variables, affective and cognitive responses, and executive functioning. Within this framework, personality traits influence how individuals perceive and respond to internal and external stimuli, shaping emotion regulation and decision-making processes. These affective and cognitive responses may increase cue reactivity and craving, particularly when rewarding online experiences provide immediate gratification or relief from negative emotions. As these reinforcing experiences are repeated, people with impaired executive control become less able to inhibit maladaptive behaviors, which increases their likelihood of developing and maintaining problematic behaviors such as PSMU and IGD.

The I-PACE model further proposes that common mechanisms underlying different problematic online behaviors may increase people’s vulnerability to experiencing more than one such behavior simultaneously (Brand et al., 2019, 2025). Accordingly, studies examining the co-occurrence of behavioral addictions suggest that certain personality traits and coping strategies—including low conscientiousness, high impulsiveness, high social anxiety, poor attachment style, and avoidant coping strategies—may be associated with vulnerability to multiple addictive behaviors (Burleigh et al., 2019). Similarly, a preference for immediate small rewards over larger delayed rewards (delay discounting) also appears to be a common factor across various behavioral addictions (Weinsztok et al., 2021). Immediate reward feedback, intermittent reinforcement schedules and, in many cases, social reinforcement processes (e.g., peer interaction in gaming, or social validation in social networking sites, SNS) may increase the likelihood that people vulnerable to one form of problematic use also engage excessively in the other (Kato et al., 2023). However, although several studies have identified associations between different behavioral addictions, these behaviors do not necessarily appear to overlap significantly or share common explanatory mechanisms (Gomez et al., 2023). Therefore, further research is needed to clarify both the similarities and differences among problematic online behaviors, as well as the factors associated with their co-occurrence.

In the present study, factors conceptually related to the dimensions proposed by the I-PACE model were examined to investigate psychosocial characteristics associated with PSMU, IGD, and the co-occurrence of both behaviors in young adults, from both a dimensional and categorical perspective. Within the person dimension, Big Five personality traits (Costa & McCrae, 1992) have been consistently associated with behavioral addictions. Higher neuroticism and lower conscientiousness are common correlates of both PSMU and IGD (Bouna-Pyrrou et al., 2018; Meynadier et al., 2024). Additionally, people exhibiting PSMU or IGD often score lower on conscientiousness and agreeableness, indicating difficulties in planning and self-regulation, a tendency to avoid negative outcomes and more aggressive and hostile behavior, leading to more interpersonal conflicts (P. K. Chew, 2022; Meynadier et al., 2024; Şalvarlı & Griffiths, 2019). However, evidence also suggests disorder-specific profiles. In this regard, high neuroticism and low openness to experience have been associated with IGD, whereas high neuroticism and high extraversion appear more characteristic of PSMU (Wartberg et al., 2023). Overall, the evidence concerning personality factors in behavioral addictions remains inconclusive.

Regarding affective factors, difficulties in emotion regulation, particularly difficulties in goal-directed behavior, have been linked to both PSMU and IGD (Marino et al., 2023; Schettler et al., 2023). Young adults experiencing difficulties in emotion regulation frequently use gaming or SNS to regulate or escape negative emotional states (Estupiñá et al., 2024; Iannattone et al., 2024; León-Méndez et al., 2026). Nevertheless, recent evidence suggests partially distinct emotion regulation profiles, with IGD showing stronger associations with limited access to adaptive strategies and emotional acceptance. However, PSMU appears more closely related to goal-directed behavior and impulse control difficulties (Lin et al., 2023; Yao et al., 2026). Collectively, these findings indicate that distinct patterns of emotion regulation difficulties may differentiate individuals with elevated IGD symptomatology from those with elevated PSMU symptomatology.

Within the cognitive dimension, decision-making styles play an important role (Scott & Bruce, 1995). Difficulties in decision-making are associated with a tendency to prioritize immediate rewards despite potential long-term negative consequences and have consistently been associated with behavioral addictions. People with IGD tend to display more impulsive and reward-oriented decision-making patterns, prioritizing immediate gratification over delayed outcomes (S. M. Müller et al., 2023, 2025; Y. Zhou et al., 2021). In contrast, PSMU has been more frequently associated with maladaptive decision-making styles characterized by avoidance of responsibility and greater reliance on external guidance (León-Méndez et al., 2026; Wegmann et al., 2021). Such decision-making tendencies may facilitate SNS use as an easily accessible strategy for emotion regulation, distraction, or social reassurance (Eichenberg et al., 2024; Kırcaburun & Griffiths, 2018). Although decision-making difficulties have been identified in both online problematic behaviors, it remains unclear whether people with co-occurring PSMU and IGD share a common decision-making profile or whether they exhibit distinct cognitive characteristics that distinguish each problematic behavior.

On the other hand, cognitive, emotional, and behavioral processes are primarily regulated by the prefrontal cortex, alterations in which are strongly linked to various behavioral addictions (Brand et al., 2014; Li et al., 2025). Executive difficulties in problematic online behaviors have been widely documented across both behavioral tasks and self-report measures (Kessling et al., 2025; Lewin et al., 2023; Zhu et al., 2023). For instance, excessive SNS use correlates with poorer executive performance (León-Méndez et al., 2026). Furthermore, neuroscientific evidence demonstrates that young regular users experience increased cognitive effort, reduced working memory efficiency, diminished inhibitory control, and altered neural activation during executive tasks (Aitken et al., 2024). Similarly, undergraduate students with IGD show poorer executive functioning alongside heightened emotional distress and decreased heart rate variability (Chen et al., 2025). Assessing self-reported prefrontal symptomatology in daily life—specifically evaluating deficits in executive, emotional, and social behavior control—enables early detection of cognitive impairment (Pedrero-Pérez et al., 2015). Anatomically, these processes depend on distinct prefrontal sub-regions: emotional control in complex decision-making relies primarily on the ventromedial prefrontal cortex; social behavior control and interpersonal expectation-building depend on the orbitofrontal cortex; and fundamental executive functions depend on the dorsolateral prefrontal cortex (Pedrero-Pérez & Ruiz-Sánchez de León, 2022). Although functional impairments across these domains are established in isolated behavioral addictions (Pérez et al., 2018), it remains unclear whether individuals with co-occurring PSMU and IGD exhibit greater overall prefrontal symptomatology than those screened positive for a single behavior.

Despite the growing evidence regarding the correlates of PSMU and IGD, most previous studies have examined these problematic behaviors either independently or from a dimensional perspective, without specifically identifying young people who may be classified as screened positive for more specific patterns of problematic use. Furthermore, to the best of our knowledge, few studies have simultaneously examined young people presenting PSMU, IGD, or their co-occurrence within the same study. Accordingly, the current study examined the psychosocial correlates associated with PSMU and IGD in young adults, using a dimensional and categorical approach. Thus, the person, affective, cognitive and executive dimensions associated with the various problematic online behaviors could be analyzed across all participants (dimensional approach), as well as within the groups formed on the basis of screened positive for PSMU, screened positive for IGD, or presenting with co-occurring PSMU and IGD (categorical approach), in order to determine whether the contribution of the examined psychological dimensions differed across these groups. The following hypotheses were proposed:

**H1.**  *Higher neuroticism and lower conscientiousness would be associated with both higher PSMU and IGD symptomatology, whereas lower agreeableness would be specifically associated with higher IGD symptomatology and higher extraversion with higher PSMU symptomatology (P. K. Chew, 2022; Meynadier et al., 2024). In young adults with co-occurring PSMU and IGD, higher neuroticism, lower conscientiousness, lower agreeableness, and lower openness to experience would be associated with higher PSMU and IGD symptomatology.*

**H2.**  *Difficulties in emotion regulation, particularly problems maintaining goal-directed behavior, would be associated with both higher PSMU and IGD symptomatology (Estupiñá et al., 2024; Lin et al., 2023; Yao et al., 2026). In young adults with co-occurring PSMU and IGD, difficulties with different emotion regulation strategies would be associated with higher PSMU and IGD symptomatology.*

**H3.**  *Different decision-making profiles are expected for each problematic behavior, with higher IGD symptomatology being associated with a more spontaneous or impulsive decision-making style, whereas higher PSMU symptomatology would be associated with a more avoidant decision-making style (Wegmann et al., 2021; Y. Zhou et al., 2021). In young adults with co-occurring PSMU and IGD, spontaneous and avoidant decision-making styles would be associated with higher PSMU and IGD symptomatology.*

**H4.**  *Self-reported difficulties in executive and emotional control are expected to show a close association with IGD and PSMU symptomatology, whereas self-reported difficulties in social behavior control will be more strongly related to IGD symptomatology (Lewin et al., 2023; Zhu et al., 2023). In young adults with co-occurring PSMU and IGD, self-reported difficulties across executive, emotional, and social behavior control would be associated with higher PSMU and IGD symptomatology.*

**H5.**  *Young adults with co-occurring PSMU and IGD will show significantly lower conscientiousness, lower agreeableness, lower openness to experience, higher neuroticism, higher difficulties in emotion regulation and greater self-reported executive, emotional, and social functioning deficits, as well as more spontaneous and avoidant decision-making, compared with those who have IGD only or PSMU only (Burleigh et al., 2019).*

## 2. Materials and Methods

### 2.1. Participants

A total of 1586 undergraduate students participated in this study (68.9% female; Mage = 20.42; SD = 2.41; age range: 17–30 years). Participants were classified into three mutually exclusive groups according to established screening cut-off scores on the Bergen Social Media Addiction Scale (BSMAS ≥ 19) and the Internet Gaming Disorder Scale—Short Form (IGDS9-SF ≥ 16). The inclusion criteria were scores of ≥19 on the BSMAS and <16 on the IGDS9-SF for young adults who screened positive for PSMU; a score ≥16 on the IGDS9-SF and <19 on the BSMAS for young adults who screened positive for IGD; and a score ≥16 on the IGDS9-SF and ≥19 on the BSMAS for young adults with co-occurring PSMU and IGD.

Of the full sample, 285 participants (17.97%) screened positive exclusively for PSMU (Mage = 20.18, SD = 2.49, 89.8% female), 254 (16.02%) screened positive exclusively for IGD (Mage = 21.04, SD = 3.04, 65.7% male), and 169 (10.65%) screened positive for both behaviors (Mage = 20.44, SD = 3.24, 67.5% female). The remaining participants did not meet the predefined cut-off criteria for any of the three groups, and were therefore not included in the group-based analyses.

### 2.2. Instruments

Bergen Social Media Addiction Scale (BSMAS; Andreassen et al., 2016; Spanish version by Copez-Lonzoy et al., 2023) is a 6-item, 5-point Likert scale (1 = very rarely, 5 = very often) designed to assess PSMU. It is a unidimensional instrument with six indicators: salience, tolerance, mood modification, relapse or loss of control, withdrawal, and conflict or functional impairment. Scores range from 6 to 30, with higher scores indicating greater PSMU symptomatology. Although a cut-off score of 24 has been proposed in previous studies, the present study used a cut-off score of 19 based on the sensitivity and specificity analyses reported by Bányai et al. (2017). Participants scoring ≥19 in BSMAS and <16 for IGDS9-SF were classified as meeting the screening criterion for PSMU. The internal consistency of the scale in this study was α = 0.80, compared with α = 0.91 reported for the original version.

Internet Gaming Disorder Scale (IGDS9-SF; Pontes & Griffiths, 2015; Spanish version by Beranuy et al., 2020) is a 9-item, 5-point Likert scale (1 = never, 5 = very often) that assesses the severity of IGD by examining both online and offline gaming activities performed over a 12-month period. The items capture the symptoms associated with preoccupation with gaming, withdrawal symptoms, tolerance, unsuccessful attempts to reduce gaming, loss of interest in other activities, continued gaming despite problems, deception about gaming time, gaming to relieve negative moods, and jeopardizing significant relationships or opportunities. Scores range from 9 to 45, with higher scores indicating greater IGD symptomatology. In the present study, a cut-off score of 16 was used based on Severo et al. (2020). Participants scoring ≥16 in IGDS9-SF and <19 for BSMAS were classified as meeting the screening criterion for IGD. The internal consistency of the scale in this study was α = 0.89, compared with α = 0.92 reported for the original version.

NEO Five-Factor Inventory (NEO-FFI-30; Körner et al., 2008; Spanish version by Fumero & de Miguel, 2023) is a 30-item, 5-point Likert scale (0 = strongly disagree, 4 = strongly agree) that assesses five major personality dimensions: neuroticism, extraversion, openness, agreeableness, and conscientiousness. It is a reliable tool for assessing the five-factor personality model, with internal consistency coefficients ranging from α = 0.62 to α = 0.77 (Fumero & de Miguel, 2023). In the present study, the coefficients ranged from α = 0.64 (conscientiousness) to α = 0.86 (neuroticism).

Difficulties in Emotion Regulation Scale (DERS-16; Bjureberg et al., 2016; Spanish version by Espitia Correa et al., 2024) is a 16-item five-point Likert scale (1 = almost never, 5 = almost always) that assesses emotion regulation difficulties across five domains: non-acceptance of negative emotions, difficulty in goal-directed behavior, impulsive control, limited access to effective strategies, and lack of emotional clarity. Higher scores indicate greater difficulties in emotion regulation. The internal consistency coefficients range from α = 0.82 (non-acceptance of negative emotions) to α = 0.88 (impulsive control).

General Decision-Making Style (GDMS; Scott & Bruce, 1995; Spanish version by Alacreu-Crespo et al., 2019) is a 24-item, 5-point Likert scale (1 = strongly disagree, 5 = strongly agree) that assesses rational, avoidant, spontaneous, intuitive, and dependent decision-making styles. The rational style is characterized by the systematic search for information and the logical evaluation of alternatives. The intuitive style reflects a tendency to rely on feelings and instincts when making decisions, whereas the dependent style is characterized by seeking and relying on the advice and guidance of others. The avoidant style refers to the tendency to postpone or avoid decision-making whenever possible, while the spontaneous style is characterized by a sense of urgency and a preference for making decisions quickly. The original scale had an internal consistency range from α = 0.72 to α = 0.91, while in this study, it ranged from α = 0.78 (intuitive) to α = 0.93 (avoidant).

Prefrontal Symptoms Inventory (PSI; Spanish version by Pedrero-Pérez et al., 2015) is a 20-item, 5-point Likert scale (1 = never, 5 = always) that measures three dimensions: executive control deficits (relating to difficulties in decision-making and a lack of initiative, concentration, and planning), social behavior control deficits (related to inappropriate reactions or comments in social contexts), and emotional control deficits (related to irritability, emotional instability, or emotional lability). Higher scores are indicative of a higher presence of self-reported prefrontal deficits or symptoms. Internal consistency coefficients range from α = 0.76 (social behavior control deficits) to α = 0.86 (executive control deficits).

### 2.3. Procedure

The data were collected through online questionnaires distributed to university students from various academic programs at the University of La Laguna (Spain). The assessment protocol included the research objectives, respondent’s informed consent, and self-report measures. The questionnaires were completed in approximately 15 min. All participants provided complete data in the questionnaires, as the assessment protocol was designed so that participants could not submit without answering every item in the questionnaires; consequently, there were no missing data. Some participants may have decided to leave the study, but the design of the assessment protocol did not allow for the quantification of dropouts. No financial or academic compensation was provided for participation. The study was conducted in accordance with the ethical principles of the Declaration of Helsinki and received approval from the Ethics Committee of the University (CEIBA2023-3331).

### 2.4. Data Analysis

First, a multivariate General Linear Model was performed on the entire sample (*N* = 1586) to evaluate associations between person, affective, cognitive, and executive dimensions and symptoms of both PSMU (BSMAS) and IGD (IGDS9-SF) jointly, from a dimensional approach. Participants were then categorized into three mutually exclusive groups based on established screening cut-off scores: screened positive for PSMU (*n* = 285), screened positive for IGD (*n* = 254), and co-occurring PSMU and IGD (*n* = 169). Within each group, partial correlations controlling for gender were conducted to examine associations between I-PACE dimensions and PSMU and IGD symptomatology. Subsequently, hierarchical linear regressions were conducted for each subgroup to identify predictors of problematic behaviors. Predictors were entered in five sequential blocks through a stepwise selection method (with an inclusion criterion of *p* ≤ 0.05 and an exclusion criterion of *p* ≥ 0.10): gender was entered in block 1, person variables in block 2, emotion regulation variables in block 3, decision-making variables in block 4, and executive functioning variables in block 5. Assumptions of linearity, homoscedasticity, normality and independence of residuals, and absence of multicollinearity were verified prior to model estimation. Finally, a multivariate analysis of covariance (MANCOVA), controlling for gender, was conducted to compare the three screening-defined groups across all I-PACE dimensions. Statistical analyses were performed using IBM SPSS Statistics (Version 26.0).

## 3. Results

### 3.1. Multivariate General Linear Model

A Multivariate General Linear Model was conducted in the entire sample with person, affective, cognitive, and executive dimensions as independent variables, and both PSMU (BSMAS) and IGD (IGDS9-SF) as dependent variables, with gender entered as a fixed factor. Preliminary analyses indicated that the assumption of homogeneity of covariance matrices was violated (Box’s M = 15.488, F = 5.154, *p* < 0.001). Therefore, results were interpreted using Pillai’s Trace, which is more robust to violations of this assumption. The analysis revealed a significant main effect for gender [Pillai’s Trace = 0.060, F (2, 1564) = 50.257, *p* < 0.001, ηp2 = 0.060].

At the multivariate level, significant associations were observed between the independent variables and both problematic behaviors (Table 1), specifically for gender (β = −0.22, *p* < 0.001 for BSMAS; β = 0.24, *p* < 0.001 for IGDS9-SF), conscientiousness (β = −0.13, *p* < 0.001 for BSMAS; β = −0.09, *p* = 0.001 for IGDS9-SF), difficulties in goal-directed behavior (β = 0.16, *p* < 0.001 for BSMAS; β = 0.08, *p* < 0.05 for IGDS9-SF), non-acceptance of negative emotions (β = 0.16, *p* < 0.05 for BSMAS; β = 0.07, *p* < 0.05 for IGDS9-SF), emotional control deficits (β = 0.06, *p* < 0.05 for BSMAS; β = −0.08, *p* < 0.01 for IGDS9-SF), and executive control deficits (β = 0.16, *p* < 0.001 for BSMAS; β = 0.18, *p* < 0.001 for IGDS9-SF). Additionally, extraversion (β = 0.14, *p* < 0.001), limited access to effective emotion regulation strategies (β = −0.10, *p* < 0.01), and dependent decision-making style (β = 0.05, *p* < 0.05) were related to BSMAS, whereas agreeableness (β = −0.09, *p* = 0.001), impulse control difficulties (β = 0.07, *p* < 0.05), and social behavior control deficits (β = 0.08, *p* < 0.01) were related to IGDS9-SF.

### 3.2. Correlational Analysis

Correlation analyses were conducted separately for young adults who screened positive for PSMU, young adults who screened positive for IGD, and young adults with co-occurring PSMU and IGD (see Supplementary Material). To minimize the likelihood of Type I error, a Bonferroni adjustment for multiple comparisons was implemented. Distinct patterns of associations emerged across the three groups, particularly for personality traits, emotion regulation, and executive functioning variables.

In young adults who screened positive for PSMU, PSMU was positively associated with neuroticism (r = 0.19, *p* < 0.001), difficulties in goal-directed behavior (r = 0.22, *p* < 0.001), non-acceptance of negative emotions (r = 0.23, *p* < 0.001), and deficits in executive (r = 0.28, *p* < 0.001) and emotional control (r = 0.21, *p* < 0.001). Similarly, in young adults who screened positive for IGD, IGD was positively associated with neuroticism (r = 0.24, *p* < 0.001), difficulties in goal-directed behavior (r = 0.27, *p* < 0.001), impulse control difficulties (r = 0.23, *p* < 0.001), limited access to effective emotion regulation strategies (r = 0.23, *p* < 0.001), and deficits in social (r = 0.25, *p* < 0.001) and executive control (r = 0.29, *p* < 0.001). In contrast, IGD was negatively associated with agreeableness (r = −0.20, *p* < 0.001) and conscientiousness (r = −0.15, *p* < 0.001). In young adults with co-occurring PSMU and IGD, PSMU and IGD scores were positively correlated (r = 0.28, *p* < 0.001). Moreover, both PSMU and IGD were positively associated with neuroticism (r = 0.25, *p* < 0.001 for both), difficulties in goal-directed behavior (r = 0.35, *p* < 0.001 and r = 0.31, *p* < 0.001, respectively), and executive control deficits (r = 0.33, *p* < 0.001 and r = 0.22, *p* < 0.001, respectively). Both types of problematic behaviors were also negatively associated with agreeableness (PSMU: r = −0.29, *p* < 0.001; IGD: r = −0.24, *p* < 0.001).

### 3.3. Hierarchical Regression Analyses

Prior to the regression analyses, the assumptions of linearity, homoscedasticity, normality of residuals, independence of residuals, and absence of multicollinearity were evaluated. Visual inspection of residual plots and normal probability (P–P) plots supported the assumptions of linearity, homoscedasticity, and normality. Durbin–Watson statistics indicated independent residuals for young adults classified as screened positive for PSMU (2.093), those classified as screened positive for IGD (2.148), and for young adults with co-occurring PSMU and IGD, with independent residuals for PSMU (1.904) and IGD (1.883) models. There was no evidence of multicollinearity, with tolerance values ranging from 0.536 to 1.000 and variance inflation factor (VIF) values ranging from 1.00 to 1.87 across all regression models.

To identify the I-PACE dimensions associated with PSMU and IGD symptomatology, hierarchical regression analyses were conducted separately for each of the three groups: young adults classified as screened positive for PSMU, young adults classified as screened positive for IGD, and young adults with co-occurring PSMU and IGD. Within each block, a stepwise selection procedure was applied to retain only associations that significantly improved model fit. Table 2 and Table 3 summarize the variables retained at each block and the final regression models. Although gender had been included in block 1, this variable was not significant in any of the models. In young adults classified as screened positive for PSMU, neuroticism was significantly associated with higher PSMU symptomatology in step 1, explaining 3% of the variance in PSMU (adjusted R^2^ = 0.03, β = 0.19, *p* < 0.01). Difficulties in goal-directed behavior entered the model in step 2, increasing the explained variance to 6% (ΔR^2^ = 0.03), followed by spontaneous decision-making style in step 3 (adjusted R^2^ = 0.07, ΔR^2^ = 0.02). Executive control deficits entered the model in step 4, explaining an additional 2% of the variance (adjusted R^2^ = 0.09, ΔR^2^ = 0.02). In the final model [F (4, 280) = 8.294, *p* < 0.001, R^2^ = 0.09], executive control deficits (β = 0.18, *p* < 0.01) were the only variable associated with greater PSMU symptomatology within the group of young people classified as screened positive for PSMU, whereas neuroticism, difficulties in goal-directed behavior, and spontaneous decision-making style no longer made unique significant contributions. VIF ranged from 1.00 to 1.41, indicating no evidence of multicollinearity.

For the young adults classified as screened positive for IGD, neuroticism was significantly associated with higher IGD symptomatology in step 1, explaining 5% of the variance in IGD (adjusted R^2^ = 0.05, β = 0.24, *p* < 0.001). Conscientiousness entered the model in step 2, increasing the explained variance to 8% (ΔR^2^ = 0.03), followed by agreeableness in step 3 (adjusted R^2^ = 0.11, ΔR^2^ = 0.03). Difficulties in goal-directed behavior entered the model in step 4, explaining an additional 2% of the variance (adjusted R^2^ = 0.13, ΔR^2^ = 0.02), whereas intuitive decision-making style entered in step 5, accounting for a further 2% of the variance (adjusted R^2^ = 0.15, ΔR^2^ = 0.02). In the final model [F (5, 248) = 8.806, *p* < 0.001, R^2^ = 0.13], lower conscientiousness (β = −0.14, *p* < 0.05), lower agreeableness (β = −0.14, *p* < 0.05), greater difficulties in goal-directed behavior (β = 0.17, *p* < 0.05), and lower intuitive decision-making style (β = −0.15, *p* < 0.05) were associated with greater IGD symptomatology. Neuroticism was no longer significantly associated with IGD symptomatology. VIF ranged from 1.00 to 1.53, indicating no evidence of multicollinearity.

In the young adult group with co-occurring PSMU and IGD, hierarchical regression analyses were conducted separately for PSMU and IGD. Lower agreeableness was significantly associated with PSMU symptomatology in step 1, explaining 8% of the variance (adjusted R^2^ = 0.08, β = −0.29, *p* < 0.001). Openness entered the model in step 2, increasing the explained variance to 13% (ΔR^2^ = 0.05), followed by neuroticism in step 3 (adjusted R^2^ = 0.16, ΔR^2^ = 0.04). Difficulties in goal-directed behavior entered the model in step 4, explaining an additional 5% of the variance (adjusted R^2^ = 0.20, ΔR^2^ = 0.05), whereas dependent decision-making style entered in step 5, accounting for a further 3% of the variance (adjusted R^2^ = 0.22, ΔR^2^ = 0.03). In the final model [F (5, 163) = 10.638, *p* < 0.001, R^2^ = 0.22], greater difficulties in goal-directed behavior (β = 0.31, *p* < 0.001), lower agreeableness (β = −0.18, *p* < 0.05), lower openness to experience (β = −0.24, *p* < 0.001), and a more dependent decision-making style (β = 0.17, *p* < 0.05) were associated with higher PSMU symptomatology. Neuroticism was no longer significantly associated with PSMU symptomatology. VIF ranged from 1.00 to 1.87, indicating no evidence of multicollinearity.

Neuroticism was significantly associated with higher IGD symptomatology in step 1, explaining 6% of the variance (adjusted R^2^ = 0.06, β = 0.25, *p* < 0.01). Openness, conscientiousness, and agreeableness entered the model in steps 2–4, increasing the explained variance to 13%, whereas difficulties in goal-directed behavior entered in step 5, accounting for an additional 2% of the variance (adjusted R^2^ = 0.15, ΔR^2^ = 0.02). In the final model [F (5, 163) = 6.697, *p* < 0.001, R^2^ = 0.15], lower openness to experience (β = −0.16, *p* < 0.05), lower agreeableness (β = −0.17, *p* < 0.05), and greater difficulties in goal-directed behavior (β = 0.19, *p* < 0.05) were associated with a higher IGD symptomatology. Neuroticism and conscientiousness were no longer significantly associated with IGD symptomatology. VIF ranged from 1.00 to 1.87, indicating no evidence of multicollinearity.

### 3.4. Multivariate Analysis of Covariance

A multivariate analysis of covariance (MANCOVA), controlling for gender, was conducted to examine the differences between three groups (screened positive for PSMU, screened positive for IGD, and with co-occurring PSMU and IGD), across the person, affective, cognitive, and executive dimensions. Preliminary analyses indicated that the assumption of homogeneity of covariance matrixes was violated (Box’s M = 1197.98, F = 1.249, *p* < 0.001). Therefore, multivariate results were interpreted through Pillai’s Trace, which is more robust to violations of this assumption. The assumption of homogeneity of error variances was also violated for some dependent variables, including BSMAS, IGDS, non-acceptance of emotional responses, and avoidant and intuitive decision-making styles, but was supported for the remaining variables. Accordingly, univariate findings for variables showing heterogeneity of variance were interpreted cautiously. Pairwise comparisons of estimated marginal means were adjusted with the Bonferroni correction to account for multiple comparisons. The multivariate analysis revealed a significant effect of group membership on the combined dependent variables (Pillai’s Trace = 0.164, F (36, 1382) = 3.432, *p* < 0.001, ηp^2^ = 0.082). Gender also showed a significant multivariate effect (Pillai’s Trace = 0.186, F (18, 690) = 8.784, *p* < 0.001, ηp^2^ = 0.186).

Univariate analysis showed significant group differences in personality variables, including neuroticism, extraversion, agreeableness, and conscientiousness. Significant group differences were also observed for all emotion regulation variables, including emotional clarity, difficulties in goal-directed behavior, limited access to emotion regulation strategies, impulse control difficulties, and non-acceptance of emotional responses. Regarding cognitive variables, significant group differences were found for avoidant, and spontaneous decision-making styles. Finally, significant differences were found across all self-reported executive functioning dimensions, including social behavior control, emotional control, and executive control deficits (see Table 4).

Bonferroni-adjusted pairwise comparisons indicated that young adults with co-occurring PSMU and IGD showed higher neuroticism, lower conscientiousness, greater executive, emotional, and social behavior control deficits, greater difficulties in all emotion regulation strategies, and a greater tendency to use avoidant and spontaneous decision-making styles than participants classified as screened positive for PSMU and those classified as screened positive for IGD. Furthermore, compared with participants classified as screened positive for PSMU, young adults with co-occurring PSMU and IGD exhibited lower agreeableness. Finally, comparisons between participants classified as screened positive for PSMU and those classified as screened positive for IGD revealed that the PSMU group exhibited both higher extraversion and greater emotional control deficits than the IGD group.

## 4. Discussion

This study examined shared and distinct person, affective, cognitive, and executive dimensions associated with PSMU and IGD symptomatology, and their co-occurrence based on the I-PACE framework (Brand et al., 2019, 2025) from a dimensional and categorical perspective. In addition to analyzing the psychosocial correlates associated with IGD and PSMU from a dimensional perspective, the participants were classified into screened positive PSMU group, screened positive IGD group, and a co-occurring PSMU and IGD group. Although previous studies have examined these problematic online behaviors separately, considerably less is known about the extent to which they share psychological correlates from a dimensional perspective or are characterized by distinct patterns from a categorical perspective. Overall, the findings indicated that the variables retained in the final regression models differed across dimensional and categorical perspectives. Specifically, the co-occurring group exhibited a more maladaptive pattern of behavior across multiple I-PACE dimensions than either single-behavior group.

The first hypothesis received partial support. From a dimensional perspective, PSMU and IGD were associated with lower conscientiousness, whereas a positive relationship was found between extraversion and PSMU, and a negative relationship between agreeableness and IGD in line with previous studies (Şalvarlı & Griffiths, 2019). Personality characteristics showed different patterns of association across PSMU, IGD, and their co-occurrence groups. Although neuroticism was positively associated with both PSMU and IGD at the bivariate level, these associations were no longer significant with either behavior when affective, cognitive, and executive dimensions were considered simultaneously. This finding suggests that the association between neuroticism and these problematic online behaviors may overlap with more immediate self-regulatory processes. In contrast, lower conscientiousness and lower agreeableness remained independently associated with greater IGD symptomatology among young adults classified as screened positive for IGD. Lower conscientiousness reflects difficulties with self-discipline, persistence, organization, and behavioral regulation over time, whereas lower agreeableness is associated with reduced empathy and cooperation and greater interpersonal antagonism or competitiveness (Costa & McCrae, 1992). These characteristics may be particularly relevant in gaming contexts that provide opportunities for achievement, competition, and status attainment, while potentially requiring fewer forms of face-to-face social interaction. This tentative interpretation is consistent with evidence linking IGD to competitiveness, achievement-oriented motives, and interpersonal conflict (Dilawar et al., 2022; Larrieu et al., 2022), as well as with evidence of lower conscientiousness and agreeableness among adult gamers (P. K. H. Chew et al., 2025). By contrast, personality traits played a more limited role among participants classified as screened positive for PSMU. One possible explanation for the absence of a clear personality profile among young adults classified as screened positive for PSMU may relate to the increasingly central role of SNS. SNS provide accessible opportunities to fulfill social and emotional needs, including a sense of belonging and interpersonal connection. Consistent with this interpretation, previous studies suggest that PSMU may be driven by both the need to belong and escapism motives (Miranda et al., 2023). In contrast, in young adults with co-occurring PSMU and IGD, lower agreeableness and lower openness to experience were associated with higher PSMU and IGD symptomatology. Lower openness reflects greater cognitive rigidity and a preference for familiar routines, whereas lower agreeableness reflects reduced interpersonal cooperation and empathy (Costa & McCrae, 1992). Together, these characteristics may be associated with reduced psychological flexibility and greater reliance on familiar, immediately rewarding forms of online engagement. In the case of PSMU, one possible, though as yet unproven, explanation could be that algorithmic personalization, which is widely used in these contexts, is reinforcing the tendency to repeatedly expose users to familiar and emotionally congruent content (Eg et al., 2022). Although the present study has not analyzed the interaction between the various psychosocial dimensions examined, within the I-PACE model this relationship between personal vulnerabilities and affective and cognitive processes could explain the persistence of more than one problematic online behavior (Brand et al., 2025).

The second hypothesis received partial support. From a dimensional perspective, PSMU and IGD were associated with difficulties in goal-directed behavior, as well as with non-acceptance of negative emotions. Other emotion regulation strategies were also associated with PSMU, such as limited access to effective emotion regulation strategies, and with IGD, such as impulse control difficulties, although previous studies have found that impulse control difficulties are associated with PSMU (Yao et al., 2026). From a categorical perspective, difficulties in goal-directed behavior were associated with IGD in young adults classified as screened positive for IGD and in young adults with co-occurring PSMU and IGD. Goal-directed behavior refers to the ability to pursue longer-term objectives despite negative emotional states (Gratz & Roemer, 2004). One possible explanation is that difficulties in this domain may increase the likelihood that immediate, rewarding online activities displace ongoing responsibilities or longer-term goals, particularly when individuals experience emotional distress. This interpretation is consistent with the incentive sensitization theory (Berridge & Robinson, 2016), according to which repeated exposure to reward-related cues may increase their motivational salience and strengthen wanting-related processes. Previous research suggests a wanting–liking dissociation in regular gamers which becomes more severe as the addiction progresses (Ma et al., 2024). Similarly, neuroimaging studies suggest that young adults with IGD rely more heavily on habitual, reward-based control than on goal-directed control (Lei et al., 2025). In young adults with co-occurring PSMU and IGD, difficulties maintaining goal-oriented behavior in the presence of negative emotions may indicate that the demands of the environment exceed the available resources. From an emotion regulation perspective (Gross, 2015), this pattern may increase reliance on strategies that provide immediate relief from unpleasant emotional states. In this regard, SNS, through interpersonal validation, and video games, through competition and achievements, may be reinforcing these online behaviors in young people. The co-occurrence of PSMU and IGD may reflect, at least in part, a broader reliance on digital activities as emotion regulation strategies. This interpretation is consistent with transdiagnostic approaches, which propose that addictive behaviors may represent different manifestations of partially shared psychological processes, rather than entirely independent categories (Perales et al., 2020).

The third hypothesis was not supported. Neither spontaneous nor avoidant decision-making was associated with PSMU or IGD among young adults classified as screened positive for PSMU or IGD. From a dimensional approach, a positive association was found only between PSMU and a dependent decision-making style. From a categorical approach, a lower tendency to use an intuitive decision-making style was associated with higher IGD symptomatology. A low intuitive decision-making style implies difficulties in rapidly interpreting internal, physical, and social cues, as well as reduced confidence in one’s own judgments (Scott & Bruce, 1995). Accordingly, the present findings may indicate that young adults classified as screened positive for IGD are less able to rely on internal signals when regulating their gaming behavior, potentially limiting their capacity to make decisions that reflect their long-term needs rather than immediate reward. This interpretation is consistent with the incentive salience theory (Berridge & Robinson, 2016), which proposes that repeated exposure to rewarding stimuli progressively enhances salience and promotes habitual behavioral responses. Consequently, the salience of gaming-related incentives over other personally relevant goals would reinforce persistent gaming behavior (W. R. Zhou et al., 2021). Additionally, a more dependent decision-making style was also associated with greater PSMU symptomatology among young adults with co-occurring PSMU and IGD, as was also observed for PSMU in the dimensional perspective. Reliance on external guidance and reassurance may increase sensitivity to social feedback and interpersonal validation, processes that are particularly salient in SNS environments (Wegmann et al., 2021). Such processes may be relevant to the association between dependent decision-making and PSMU symptomatology in this group. Overall, the findings suggest that decision-making styles may capture only part of the cognitive processes relevant to problematic online behaviors. Future studies should therefore examine more specific processes, including reward-based decision-making, delay discounting, risk sensitivity, and decision-making under emotional distress.

The fourth hypothesis received partial support. Results confirmed executive and emotional control deficits for both IGD and PSMU and social behavior control deficits for IGD, from a dimensional perspective, as suggested by other studies (Lewin et al., 2023; Zhu et al., 2023). However, from a categorical perspective, only executive control deficits were associated with higher PSMU symptomatology in young adults classified as screened positive for PSMU, but not with IGD symptomatology in young adults classified as screened positive for IGD. Deficits in executive functions have consistently been associated with PSMU, particularly through poorer inhibitory control and attentional impulsivity that may facilitate excessive engagement with SNS (S. M. Müller et al., 2021). More broadly, neuroimaging research has implicated frontal systems involved in executive functioning across behavioral addictions, which supports the relevance of impaired self-regulation to problematic online behaviors (León-Méndez et al., 2024; Lewin et al., 2023; Zhu et al., 2023). Unexpectedly, executive control was not associated with IGD symptomatology when the other I-PACE dimensions were considered. One possible explanation is that executive control may overlap with other affective and cognitive processes included in the models. In fact, lower intuitive decision-making was associated with higher IGD symptomatology, suggesting that difficulties in intuitive decision-making may be particularly relevant to the regulation of gaming behavior and the identification of cues that could support behavioral self-regulation. Notably, young adults with co-occurring PSMU and IGD showed difficulties across executive, emotional, and social behavior control. The presence of greater difficulties in the co-occurring group does not necessarily imply that these variables are associated with higher symptomatology within that group; rather, group differences and within-group associations address different questions and may therefore yield different patterns of findings.

Finally, the fifth hypothesis was partially supported. As expected, young adults with co-occurring PSMU and IGD exhibited significantly higher neuroticism, lower conscientiousness, greater emotion regulation difficulties, greater self-reported prefrontal symptoms, and a higher tendency toward avoidant and spontaneous decision-making styles compared with young adults in both single-behavior groups. However, differences in low agreeableness were found only between young adults with co-occurring PSMU and IGD and the PSMU group, but did not differ significantly from the IGD group. Previous research suggests that lower agreeableness is more consistently associated with IGD than with PSMU (P. K. Chew, 2022; Meynadier et al., 2024). The fact that the group that screened positive for PSMU scored higher on agreeableness may also be explained by the role of SNS in facilitating or promoting interpersonal relationships among young people, in contrast to the IGD group, where competitiveness may be more prominent. Furthermore, the expected differences between the groups in openness to experience were not confirmed. This finding is also broadly consistent with previous evidence indicating that openness has a limited and inconsistent association with problematic digital behaviors. In particular, previous research has found no significant association between openness and IGD (P. K. Chew, 2022), while evidence concerning PSMU suggests only a very small association, with limited incremental contribution beyond other personality dimensions, such as neuroticism and conscientiousness (Meynadier et al., 2024). Accordingly, openness to experience may have limited discriminative value for distinguishing between different forms of problematic digital engagement. As expected, emotion regulation difficulties and executive, social, and emotional control difficulties were greater in the young adults with co-occurring PSMU and IGD than in the other two groups, which presented only one type of problematic behavior. These findings may highlight the role that various forms of problematic online behavior may play as a means of attempting to escape or avoid confronting emotional difficulties (Estupiñá et al., 2024), which, in turn, may limit emotional, executive, and social control perception (Pérez et al., 2018). Previous research has similarly suggested that behavioral coping strategies, including avoidance and attempts to compensate for unsatisfactory interpersonal relationships, may contribute to the maintenance of co-occurring problematic behaviors (Vollmer et al., 2014). Furthermore, previous evidence indicates that the co-occurrence of problematic online behaviors is associated with greater psychological distress and functional impairment than either behavior alone (Pontes, 2017; Monacis et al., 2017).

Taken together, young adults with co-occurring PSMU and IGD exhibited a profile that may be associated with greater difficulties in the person, executive functioning, emotion regulation, and decision-making dimensions. However, except for executive control, the effect sizes were small. These findings should therefore be interpreted with caution. The cross-sectional design also prevents conclusions regarding the directionality of these relationships. Nevertheless, the convergence of difficulties across emotion regulation, executive control, and decision-making may be consistent with models suggesting that problematic online behaviors are partly characterized by impaired self-regulation and greater reliance on immediately rewarding or habitual responses at the expense of flexible, goal-directed regulation (Brand et al., 2019, 2025; Koob & Volkow, 2016).

### 4.1. Limitations and Future Research

The findings should be interpreted in light of several limitations. First, the sample consisted exclusively of university students and was predominantly female, limiting the generalizability of the findings. Future studies should include more diverse populations across different age groups and cultural contexts. Second, gender distribution differed markedly across groups, and residual confounding may remain despite statistical adjustment. Third, participants were classified according to validated screening cut-offs rather than clinical diagnoses, which may have over- or underestimated the prevalence of problematic behaviors. Nevertheless, studying young adults who are classified as screened positive for problematic online behaviors may help identify psychological characteristics associated with problematic use before more serious clinical manifestations emerge. Fourth, because variables were selected using a stepwise procedure, the specific sets of variables retained in the final models require cross-validation in independent samples. Fifth, because all analyses were cross-sectional and based on self-report measures, the directionality and clinical significance of the observed associations cannot be established. Longitudinal studies are needed to clarify the factors involved in the onset, maintenance, and progression of PSMU, IGD, and their co-occurrence. Finally, the variables examined explained a relatively modest proportion of the variance in PSMU and IGD, indicating that additional factors, such as self-esteem, coping motives, or social reward sensitivity, should be incorporated into future models (Schivinski et al., 2020).

### 4.2. Theoretical and Clinical Implications

Despite these limitations, the findings have several theoretical implications. First, approaching the psychosocial correlates associated with problematic online behaviors from a dimensional or categorical perspective has significant theoretical implications. From a dimensional perspective, the assumption is that these behaviors are distributed along a continuum at the extreme end of which they can become dysfunctional; however, identifying the point on the continuum at which a behavior moves from being adaptive to maladaptive is critical. From a categorical perspective, these behaviors would be understood as discrete and qualitatively distinct categories, for which establishing a cut-off point is crucial. This perspective may facilitate a therapeutic approach that is more focused on the specific problem and the individual. Second, the results point to distinct psychological profiles across the three screening-defined groups partially supporting the I-PACE framework, which suggests that PSMU and IGD are associated with both shared and differentiated psychological characteristics. Therefore, PSMU and IGD should not necessarily be regarded as interchangeable manifestations of a single underlying condition, although the value of examining common psychological processes across different problematic online behaviors is highlighted. However, further studies are needed to examine in depth the role played by the interaction between the different dimensions of the model, beyond the simple additive effect analyzed in this study. Third, the results suggest that the co-occurrence of PSMU and IGD may represent a less adaptive phenotype characterized by the accumulation of risk factors across multiple domains (person, emotion regulation, and self-reported prefrontal symptomatology), rather than simply the sum of two independent problematic behaviors, consistent with dimensional models of psychopathology. Finally, the differential contribution of personality traits, emotion regulation, executive functioning, and decision-making highlights the potential importance of considering the interplay between relatively stable predispositions and more dynamic cognitive–affective processes when conceptualizing problematic online behaviors, as proposed by the I-PACE model.

This study highlights the need for further research into the relationship between person, affective, cognitive, and executive dimensions and problematic online behaviors involving gaming and SNS. The results suggest different clinical implications for young people who engage in SNS and online gaming, depending on whether their use is controlled or excessive. The present findings may contribute to the development of new hypotheses, for example, regarding the role of extraversion in adaptive SNS use, as opposed to executive control as a potential risk factor for PSMU. Likewise, while impulse control difficulties may be a risk factor among young people who are beginning to engage in gaming, difficulties in maintaining goal-directed behavior may represent a risk factor for those who show excessive use. Therefore, further research is needed to clarify how person, affective, cognitive, and executive dimensions contribute to problematic online behaviors, as well as to guide the development of future clinical interventions aimed at addressing and preventing these types of problematic online behaviors. In this regard, future interventions should be tailored to young people’s needs and their potential risk of developing a problematic online behavior or behavioral addiction. Based on our findings, interventions aimed at strengthening executive control strategies may represent a useful starting point for young people screened positive for PSMU. In contrast, fostering conscientiousness and agreeableness, together with promoting goal-setting skills and more intuitive decision-making strategies, may help reduce the risk of IGD. Future research should also examine young people who present with co-occurring problematic online behaviors, with particular attention to the role of openness to experience and difficulties in goal-setting as potential risk factors.

## 5. Conclusions

The present findings revealed that PSMU and IGD were associated with partially distinct psychological profiles both from a dimensional and a categorical perspective, although the study included a selection of constructs that are conceptually related to the I-PACE model. Specifically, young adults classified as screened positive for PSMU were characterized by executive control deficits, whereas those classified as screened positive for IGD showed lower agreeableness and conscientiousness, greater difficulties in goal-directed behavior, and a lower tendency to rely on intuitive decision-making. Among young adults with co-occurring PSMU and IGD, lower agreeableness and openness to experience and greater difficulties maintaining goal-directed behavior were associated with greater symptomatology of both problematic behaviors. Comparisons across the three groups further indicated that young adults with co-occurring PSMU and IGD exhibited differences in several psychosocial dimensions examined, including personality, emotion regulation, decision-making, and self-reported prefrontal symptomatology. These findings suggest that co-occurring PSMU and IGD may be associated with a more maladaptive pattern of functioning than the mere simultaneous presence of two problematic behaviors. Additionally, the present findings highlight the need for targeted prevention and early intervention strategies to reduce the risk of more severe presentations of these problematic behaviors.

## Figures and Tables

**Table 1:** Multivariate General Linear Model of person, affective, cognitive, and executive dimensions in the entire sample.

	BSMAS						IGDS9-SF					
	B	SE	β	t	*p*	ηp^2^	B	SE	β	t	*p*	ηp^2^
Gender	−2.481	0.278	−0.219	−8.921	<0.001	0.048	3.196	0.327	0.244	9.759	<0.001	0.057
NEO-FFI-30												
Neuroticism	0.027	0.031	0.031	0.876	0.381	0.000	0.040	0.037	0.039	1.080	0.280	0.001
Extraversion	0.167	0.030	0.139	5.500	<0.001	0.019	−0.001	0.036	−0.001	−0.028	0.977	0.000
Openness to experience	−0.021	0.024	−0.020	−0.895	0.371	0.001	−0.005	0.028	−0.004	−0.165	0.869	0.000
Agreeableness	−0.070	0.033	−0.055	−2.121	0.034	0.003	−0.131	0.039	−0.090	−3.360	0.001	0.007
Conscientiousness	−0.199	0.040	−0.131	−4.986	<0.001	0.016	−0.160	0.047	−0.091	−3.392	0.001	0.007
DERS-16												
Clarity	0.120	0.067	0.051	1.784	0.075	0.002	−0.037	0.079	−0.013	−0.463	0.644	0.000
Goals	0.267	0.054	0.162	4.923	<0.001	0.015	0.141	0.064	0.075	2.219	0.027	0.003
Impulse control	0.009	0.056	0.005	0.165	0.869	0.000	0.154	0.066	0.074	2.343	0.019	0.003
Strategies	−0.103	0.046	−0.099	−2.238	0.025	0.003	0.023	0.054	0.019	0.429	0.668	0.000
Non-acceptance	0.261	0.056	0.164	4.654	<0.001	0.014	0.131	0.066	0.072	1.988	0.047	0.003
GDMS												
Rational	−0.062	0.037	−0.043	−1.680	0.093	0.002	0.015	0.043	0.009	0.336	0.737	0.000
Intuitive	0.066	0.034	0.047	1.936	0.053	0.002	0.006	0.040	0.004	0.145	0.885	0.000
Dependent	0.064	0.030	0.052	2.144	0.032	0.003	−0.024	0.035	−0.017	−0.677	0.499	0.000
Avoidant	0.026	0.025	0.028	1.032	0.302	0.001	0.000	0.029	0.000	−0.016	0.987	0.000
Spontaneous	−0.033	0.034	−0.026	−0.962	0.336	0.001	0.011	0.041	0.007	0.268	0.789	0.000
PSI-20												
Social behavior control	−0.044	0.043	−0.027	−1.018	0.309	0.001	0.142	0.051	0.077	2.801	0.005	0.005
Emotional control	0.078	0.039	0.061	2.009	0.045	0.003	−0.120	0.046	−0.082	−2.624	0.009	0.004
Executive control	0.094	0.020	0.157	4.771	<0.001	0.014	0.127	0.023	0.182	5.448	<0.001	0.019

*Note.* B = Unstandardized regression coefficient; SE = Standard error; β = Standardized regression coefficient; ηp^2^ = Partial eta squared. PSMU: Problematic Social Media Use; IGD: Internet Gaming Disorder; Clarity: Lack of emotional clarity; Goals: Difficulties engaging in goal-directed behavior; Impulse: Impulse control difficulties; Strategies: Limited access to emotion regulation strategies; Non-acceptance: Non-acceptance of emotional responses; Rational: Rational decision-making style; Intuitive: Intuitive decision-making styles; Dependent: Dependent decision-making style; Avoidant: Avoidant decision-making style; Spontaneous: Spontaneous decision-making style.

**Table 2:** Regression model on young adults classified as vulnerable to PSMU and those classified as vulnerable to IGD.

PSMU (n = 285)							IGD (n = 254)						
Variables	R^2^ adj	ΔR	β	F	Tolerance	VIF	Variables	R^2^ adj	ΔR	β	F	Tolerance	VIF
*Step 1:*	0.03	0.03		11.121 **			*Step 1:*	0.05	0.06		14.982 ***		
Neuroticism			0.19 **		1.00	1.00	Neuroticism			0.24 ***		1.00	1.00
*Step 2:*	0.06	0.03		9.562 ***			*Step 2:*	0.08	0.03		11.999 ***		
Neuroticism			0.13 *		0.88	1.14	Neuroticism			0.26 ***		0.99	1.01
Goals			0.17 **		0.88	1.14	Conscientiousness			−0.18 ***		0.99	1.01
*Step 3:*	0.07	0.02		8.389 ***			*Step 3:*	0.11	0.03		10.563 ***		
Neuroticism			0.11		0.86	1.16	Neuroticism			0.22 ***		0.94	1.06
Goals			0.14 *		0.84	1.19	Conscientiousness			−0.19 ***		0.99	1.02
Spontaneous			0.14 *		0.92	1.09	Agreeableness			−0.16 ***		0.95	1.06
*Step 4:*	0.09	0.02		8.294 ***			*Step 4:*	0.13	0.02		9.176 ***		
Neuroticism			0.05		0.76	1.32	Neuroticism			0.14		0.67	1.49
Goals			0.11		0.10	1.24	Conscientiousness			−0.17 **		0.97	1.03
Spontaneous			0.11		0.10	1.15	Agreeableness			−0.14 *		0.92	1.09
Executive control			0.18 **		0.71	1.41	Goals			0.16 *		0.67	1.50
							*Step 5:*	0.15	0.02		8.806 ***		
							Neuroticism			0.11		0.66	1.53
							Conscientiousness			−0.14 *		0.93	1.08
							Agreeableness			−0.14 *		0.92	1.09
							Goals			0.17 *		0.66	1.51
							Intuitive			−0.15 *		0.94	1.07

*Note:* * *p* < 0.05; ** *p* < 0.01; *** *p* < 0.001. PSMU: Problematic Social Media Use; IGD: Internet Gaming Disorder; Goals: Difficulties engaging in goal-directed behavior; Intuitive: Intuitive decision-making styles; Spontaneous: Spontaneous decision-making style.

**Table 3:** Regression model on each problematic behavior in young adults with co-occurring PSMU and IGD.

Co-Occurring PSMU and IGD (n = 169)													
PSMU							IGD						
Variables	R^2^ adj	ΔR	β	F	Tolerance	VIF	Variables	R^2^ adj	ΔR	β	F	Tolerance	VIF
*Step 1:*	0.08	0.09		15.416 ***			*Step 1:*	0.06	0.06		11.093 **		
Agreeableness			−0.29 ***		1.00	1.00	Neuroticism			0.25 **		1.00	1.00
*Step 2:*	0.13	0.05		13.050 ***			*Step 2:*	0.08	0.03		8.679 ***		
Agreeableness			−0.29 ***		0.99	1.00	Neuroticism			0.27 ***		0.99	1.01
Openness to experience			−0.23 **		0.99	1.00	Openness to experience			−0.18 *		0.99	1.01
*Step 3:*	0.16	0.04		11.370 ***			*Step 3:*	0.10	0.03		7.450 ***		
Agreeableness			−0.22 **		0.89	1.13	Neuroticism			0.30 ***		0.95	1.05
Openness to experience			−0.25 **		0.98	1.02	Openness to experience			−0.17 *		0.99	1.02
Neuroticism			0.20 **		0.88	1.14	Conscientiousness			−0.16 *		0.96	1.05
*Step 4:*	0.20	0.05		11.528 ***			*Step 4:*	0.13	0.03		7.224 ***		
Agreeableness			−0.19 **		0.88	1.14	Neuroticism			0.24 **		0.86	1.17
Openness to experience			−0.24 **		0.98	1.02	Openness to experience			−0.16 *		0.98	1.02
Neuroticism			0.03		0.57	1.74	Conscientiousness			−0.18 *		0.95	1.06
Goals			0.28 **		0.60	1.67	Agreeableness			0.19 *		0.88	1.14
*Step 5:*	0.22	0.03		10.638 ***			*Step 5:*	0.15	0.02		6.697 ***		
Agreeableness			−0.18 *		0.88	1.14	Neuroticism			0.12		0.54	1.87
Openness to experience			−0.24 ***		0.98	1.02	Openness to experience			−0.16 *		0.98	1.02
Neuroticism			−0.03		0.54	1.87	Conscientiousness			−0.15		0.90	1.12
Goals			0.31 ***		0.59	1.69	Agreeableness			−0.17 *		0.86	1.16
Dependent			0.17 *		0.91	1.10	Goals			0.19 *		0.57	1.77

*Note:* * *p* < 0.05; ** *p* < 0.01; *** *p* < 0.001. PSMU: Problematic Social Media Use; IGD: Internet Gaming Disorder; Openness: Openness to experience; Goals: Difficulties engaging in goal-directed behavior; Dependent: Dependent decision-making style.

**Table 4:** Estimated marginal means, 95% confidence intervals, univariate tests, and Bonferroni-adjusted pairwise comparisons across the PSMU, IGD, and co-occurring PSMU and IGD groups.

	PSMU (n = 285) (Group 1)		IGD (n = 254) (Group 2)		Co-Occurring PSMU and IGD (n = 169) (Group 3)		F (2, 703)	*p*	ηp^2^	Bonferroni-Adjusted Pairwise Comparisons
	EMM (SE)	95% CI	EMM (SE)	95% CI	EMM (SE)	95% CI				
NEO-FFI										
Neuroticism	11.64 (0.38)	10.88–12.39	11.10 (0.35)	10.43–11.79	13.25 (0.47)	12.33–14.18	6.94	0.001	0.019	3 > 1 *; 3 > 2 **
Extraversion	13.57 (0.32)	12.95–14.19	12.35 (0.29)	11.79–12.91	13.22 (0.39)	12.46–13.98	3.85	0.022	0.011	1 > 2 *
Openness to experience	14.34 (0.32)	13.71–14.97	15.10 (0.35)	14.42–15.78	14.49 (0.39)	13.73–15.26	1.06	0.348	0.003	
Agreeableness	17.26 (0.26)	16.75–17.77	16.53 (0.28)	15.98–17.09	15.75 (0.32)	15.12–16.37	7.78	<0.001	0.022	1 > 3 ***
Conscientiousness	13.77 (0.22)	13.35–14.20	13.88 (0.23)	13.42–14.34	12.94 (0.26)	12.42–13.45	4.20	0.015	0.012	1 > 3 *; 2 > 3 *
DERS-16										
Clarity	5.89 (0.14)	5.61–6.17	5.63 (0.15)	5.33–5.93	6.28 (0.17)	5.95–6.62	7.03	0.001	0.019	3 > 1 *; 3 > 2 **
Goals	8.94 (0.20)	8.55–9.34	8.64 (0.22)	8.22–9.07	10.04 (0.24)	9.56–10.52	14.12	<0.001	0.038	3 > 1 ***; 3 > 2 ***
Impulse control	6.51 (0.19)	6.13–6.88	6.44 (0.21)	6.03–6.85	7.66 (0.23)	7.20–8.12	12.09	<0.001	0.033	3 > 1 ***; 3 > 2 ***
Strategies	12.80 (0.32)	12.17–13.43	12.56 (0.35)	11.88–13.24	14.50 (0.39)	13.73–15.26	12.10	<0.001	0.033	3 > 1 **; 3 > 2 ***
Non-acceptance	7.73 (0.22)	7.31–8.15	7.24 (0.23)	6.78–7.69	8.75 (0.26)	8.24–9.26	12.10	<0.001	0.033	3 > 1 **; 3 > 2 ***
GDMS										
Rational	19.20 (0.24)	18.73–19.67	19.44 (0.26)	18.93–19.94	18.49 (0.29)	17.92–19.06	2.39	0.093	0.007	
Intuitive	17.92 (0.25)	17.43–18.40	17.37 (0.27)	16.85–17.90	17.71 (0.30)	17.12–18.30	0.92	0.400	0.003	
Dependent	18.66 (0.27)	18.13–19.19	17.88 (0.29)	17.30–18.45	18.28 (0.33)	17.64–18.93	2.63	0.073	0.007	
Avoidant	14.10 (0.37)	13.38–14.83	13.78 (0.40)	12.99–14.56	15.92 (0.45)	15.03–16.80	7.36	<0.001	0.020	3 > 1 **; 3 > 2 **
Spontaneous	10.10 (0.26)	9.59–10.61	9.62 (0.28)	9.07–10.18	11.18 (0.32)	10.56–11.80	5.90	0.003	0.016	3 > 1 *; 3 > 2 **
ISP-20										
Executive control	29.90 (0.57)	28.79–31.07	29.75 (0.51)	28.75–30.75	35.85 (0.69)	34.50–37.20	31.21	<0.001	0.081	3 > 1 ***; 3 > 2 ***
Social behavior control	8.08 (0.24)	7.62–8.55	8.51 (0.21)	8.09–8.93	9.51 (0.29)	8.94–10.07	8.09	<0.001	0.022	3 > 1 ***; 3 > 2 *
Emotional control	11.33 (0.27)	10.81–11.85	10.31 (0.24)	9.84–10.78	12.47 (0.32)	11.83–13.10	13.94	<0.001	0.038	1 > 2 *; 3 > 1 *; 3 > 2 ***

*Note:* * *p* < 0.05; ** *p* < 0.01; *** *p* < 0.001. EMM: Estimated marginal means (EMMs) and 95% confidence intervals (CI), adjusted for gender. PSMU: Problematic Social Media Use; IGD: Internet Gaming Disorder; Clarity: Lack of emotional clarity; Goals: Difficulties engaging in goal-directed behavior; Impulse: Impulse control difficulties; Strategies: Limited access to emotion regulation strategies; Non-acceptance: Non-acceptance of emotional responses; Rational: Rational decision-making style; Intuitive: Intuitive decision-making styles; Dependent: Dependent decision-making style; Avoidant: Avoidant decision-making style; Spontaneous: Spontaneous decision-making style.

## Data Availability

The data that support the findings of this study are available from the corresponding author upon reasonable request.

## Supplementary Materials

The following supporting information can be downloaded at https://www.mdpi.com/article/10.3390/ejihpe16090141/s1, Table S1: Correlation between each problematic behavior and the I-PACE variables for the PSMU-vulnerable group. Table S2: Correlation between each problematic behavior and the I-PACE variables for the IGD-vulnerable group. Table S3: Correlation between each problematic behavior and the I-PACE variables for the co-occurrence-vulnerable group.

## Abbreviations

The following abbreviations are used in this manuscript:

PSMU	Problematic Social Media Use
IGD	Internet Gaming Disorder
SNS	Social Networking Sites
