# Supplementary material for: Person, Affective, Cognitive, and Executive Dimensions Associated with Problematic Social Media Use, Internet Gaming Disorder, and Their Co-Occurrence in Young Adults
Source: Eur J Investig Health Psychol Educ. 2026 Sep 15;16(9):141. doi: 10.3390/ejihpe16090141 (PMC13605489; doi:10.3390/ejihpe16090141)
Supplement: Supplementary file 1 [file ejihpe-16-00141-s001.zip › ejihpe-4489270-supplementary.pdf]

Table S1. Correlation between each problematic behavior and the I-PACE variables for the PSMU-vulnerable group

	1	2	3	4	5	6	7	8	9	10	11	12	13	14	15	16	17	18	19
1. BSMAS																			
2. IGDS9-sf	-.01																		
Personality traits																			
3. Neuroticism	.19**	.01																	
4. Extraversion	-.01	.07	-.10																
5.Openness	-.04	.04	-.03	.072															
6.Agreeableness	-.16	-.09	-.30**	.070	.04														
7. Conscientiousness	-.06	.00	.18	.36**	-.02	-.04													
Emotional Dysregulation																			
8. Clarity	.13	.05	.41**	-.06	-.14	-.18	.09												
9.Goals	.22**	.02	.35**	-.05	-.05	-.12	.01	.47**											
10.Impulse	.17	.00	.33**	.00	-.06	-.30**	.12	.49**	.56**										
11.Strategies	.20	.03	.55**	-.09	-.04	-.22**	.03	.62**	.68**	.62**									
12.Non-acceptance	.23**	.02	.56**	-.05	-.01	-.22**	.06	.53**	.54**	.57**	.77**								
Decision-Making																			
13. Rational	-.12	.04	-.02	.19	.15	.04	.41**	-.04	-.02	.03	-.03	-.01							
14.Intuitive	-.04	-.06	-.04	.26**	.25**	-.04	.25**	.02	.03	.02	-.03	-.02	.34**						
15. Dependent	.03	-.01	.33**	.14	-.01	.01	.22**	.14	.14	.09	.11	.13	.23**	.21**					
16. Avoidant	.13	-.01	.42**	-.02	-.03	-.13	-.05	.22**	.18	.15	.24**	.22**	-.08	.00	.33**				
17. Spontaneous	.2	.05	.22**	.10	-.04	-.25**	-.04	.24**	.26**	.19	.18	.21**	-.32**	.17	.11	.37**			
Prefrontal symptoms																			
18. Social control	.20	.08	.13	.22**	-.06	-.29**	-.05	.13	.12	.13	.10	.18	-.23**	-.05	.06	.16	.33**		
19.Emotional control	.21**	-.02	.48**	.17	-.11	-.23**	.18	.39**	.27**	.36**	.43**	.40**	-.01	.07	.17	.29**	.29**	.25**	
20.Executive control	.28**	.09	.45**	-.18	-.03	-.25**	-.19	.26**	.37**	.24**	.36**	.33**	-.24**	-.14	.22**	.52**	.33**	.33**	.30**

Note: *Statistical significance adjusted for the Bonferroni correction ($p < .001$).*

Table S2. Correlation between each problematic behavior and the I-PACE variables for the IGD-vulnerable group

	1	2	3	4	5	6	7	8	9	10	11	12	13	14	15	16	17	18	19
1. BSMAS																			
2. IGDS9-sf	.01																		
Personality traits																			
3. Neuroticism	.15	.24**																	
4. Extraversion	.13	-.14	-.12																
5. Openness	.07	-.05	-.06	.20															
6. Agreeableness	-.05	-.20**	-.22**	.07	.03														
7. Conscientiousness	-.08	-.15**	.11	.26**	.08	-.08													
Emotional Dysregulation																			
8. Clarity	.15	.13	.56**	-.15	-.08	-.25**	.11												
9. Goals	.12**	.27**	.55**	-.07	.08	-.25**	-.03	.45**											
10. Impulse	.15	.23**	.43**	-.03	-.09	-.38**	.03	.48**	.59**										
11. Strategies	.12	.23**	.72**	-.17	-.01	-.27**	.04	.61**	.69**	.60**									
12. Non-acceptance	.18	.20	.60**	-.16	-.07	-.15	.03	.51**	.51**	.41**	.76**								
Decision-Making																			
13. Rational	-.06	-.14	-.14	.07	.22**	-.01	.28**	-.06	-.05	-.17	-.09	-.08							
14. Intuitive	.05	-.20	-.11	.35**	.06	.01	.19	-.09	-.01	.02	-.10	-.17	.19						
15. Dependent	.11	.00	.30**	.12	-.04	.15	.03	.14	.14	.11	.21**	.19	.12	.19					
16. Avoidant	.05	.07	.40**	-.06	-.08	-.11	-.11	.29**	.36**	.16**	.36**	.31**	-.05	-.01	.30**				
17. Spontaneous	.07	.12	.17	.23**	-.14	-.29**	-.07	.12	.24**	.31**	.12	.10	-.29**	.32**	.10	.21			
Prefrontal symptoms																			
18. Social control	.09	.25**	.31**	-.02	-.05	-.30**	-.17	.16	.33**	.29**	.37**	.32**	-.18	-.01	.11	.26**	.30**		
19.Emotional control	.16	.12	.52**	.01	.01	-.18	.01	.42**	.39**	.51**	.57**	.48**	-.13	.12	.20	.25**	.29**	.39**	
20.Executive control	.10	.29**	.60**	-.16	-.12	-.18	-.18	.34**	.50**	.34**	.56**	.45**	-.15	-.10	.24**	.51**	.17	.43**	.40**

Note: *Statistical significance adjusted for the Bonferroni correction ($p < .001$).*

Table S3. Correlation between each problematic behavior and the I-PACE variables for the cooccurrence-vulnerable group

	1	2	3	4	5	6	7	8	9	10	11	12	13	14	15	16	17	18	19
1. BSMAS																			
2. IGDS9-sf	.28**																		
Personality traits																			
3. Neuroticism	.25**	.25**																	
4. Extraversion	.01	-.08	-.13																
5. Openness	-.23	-.15	.11	.14															
6. Agreeableness	-.29**	-.24**	-.32**	-.22	.03														
7. Conscientiousness	-.07	-.11	.20	.37**	.07	-.17													
Emotional Dysregulation																			
8. Clarity	.25	.19	.41**	-.14	-.02	-.18	.05												
9. Goals	.35**	.31**	.63**	-.08	.04	-.28**	-.04	.41**											
10. Impulse	.21	.19	.39**	-.03	.09	-.39**	.10	.47**	.56**										
11. Strategies	.19	.26	.69**	-.20	.09	-.34**	.02	.60**	.68**	.67**									
12. Non-acceptance	.17	.18	.65**	-.14	.16	-.32**	.04	.46**	.51**	.55**	.72**								
Decision-Making																			
13. Rational	.10	-.05	.06	.04	.12	-.08	.28**	-.03	-.04	-.08	-.03	.07							
14. Intuitive	-.12	-.07	-.12	.24	.21	-.02	.22	-.07	-.14	-.04	-.09	-.07	.14						
15. Dependent	.22	.08	.28**	.02	.01	-.13	.18	.12	.11	.14	.15	.18	.21	.07					
16. Avoidant	.22	.12	.41**	-.04	-.02	-.22	.01	.18	.37**	.20	.29**	.31**	.12	.07	.24				
17. Spontaneous	.09	.08	.03	.29**	.09	-.28**	-.05	.06	.13	.19*	.09	.09	-.26	.28**	.00	.25			
Prefrontal symptoms																			
18. Social control	.18	.13	.27**	.22	.12	-.43	-.01	.06	.25	.25	.30**	.32**	-.05	.02	.11	.11	.27**		
19.Emotional control	.22	.01	.40	.12	.06	-.26	.17	.35**	.41**	.53**	.52**	.40**	-.02	.04	.11	.13	.26**	.37**	
20.Executive control	.33**	.22**	.52**	-.11	-.15	-.27**	-.05	.26	.55**	.33**	.43**	.45**	.01	-.14	.18	.58**	.20	.31**	.39**

***Note**: Statistical significance adjusted for the Bonferroni correction ($p < .001$).*
